# Decoding the Influence of Obesity on Prostate Cancer and Its Transgenerational Impact

**DOI:** 10.3390/nu15234858

**Published:** 2023-11-21

**Authors:** Mariana Santos-Pereira, Sara C. Pereira, Irene Rebelo, Maria A. Spadella, Pedro F. Oliveira, Marco G. Alves

**Affiliations:** 1 iBiMED-Institute of Biomedicine and Department of Medical Science, University of Aveiro, 3810-193 Aveiro, Portugal; marianasantospereira05@gmail.com; 2 Endocrine and Metabolic Research, Unit for Multidisciplinary Research in Biomedicine (UMIB), School of Medicine and Biomedical Sciences (ICBAS), University of Porto, 4050-313 Porto, Portugal; saracatarinapereira@gmail.com; 3 Laboratory for Integrative and Translational Research in Population Health (ITR), University of Porto, 4099-002 Porto, Portugal; 4 LAQV-REQUIMTE and Department of Chemistry, Campus Universitario de Santiago, University of Aveiro, 3810-193 Aveiro, Portugal; p.foliveira@ua.pt; 5 Department of Pathology, Faculty of Medicine, University of Porto, 4200-319 Porto, Portugal; 6 UCIBIO-REQUIMTE, Laboratory of Biochemistry, Department of Biologic Sciences, Pharmaceutical Faculty, University of Porto, 4050-313 Porto, Portugal; irebelo@ff.up.pt; 7 Human Embryology Laboratory, Marília Medical School, Marília 17519-030, SP, Brazil; maspadella@gmail.com

**Keywords:** obesity, prostate cancer, transgenerational, epigenetic

## Abstract

In recent decades, the escalating prevalence of metabolic disorders, notably obesity and being overweight, has emerged as a pressing concern in public health. Projections for the future indicate a continual upward trajectory in obesity rates, primarily attributable to unhealthy dietary patterns and sedentary lifestyles. The ramifications of obesity extend beyond its visible manifestations, intricately weaving a web of hormonal dysregulation, chronic inflammation, and oxidative stress. This nexus of factors holds particular significance in the context of carcinogenesis, notably in the case of prostate cancer (PCa), which is a pervasive malignancy and a leading cause of mortality among men. A compelling hypothesis arises from the perspective of transgenerational inheritance, wherein genetic and epigenetic imprints associated with obesity may wield influence over the development of PCa. This review proposes a comprehensive exploration of the nuanced mechanisms through which obesity disrupts prostate homeostasis and serves as a catalyst for PCa initiation. Additionally, it delves into the intriguing interplay between the transgenerational transmission of both obesity-related traits and the predisposition to PCa. Drawing insights from a spectrum of sources, ranging from in vitro and animal model research to human studies, this review endeavors to discuss the intricate connections between obesity and PCa. However, the landscape remains partially obscured as the current state of knowledge unveils only fragments of the complex mechanisms linking these phenomena. As research advances, unraveling the associated factors and underlying mechanisms promises to unveil novel avenues for understanding and potentially mitigating the nexus between obesity and the development of PCa.

## 1. Introduction

The prevalence of metabolic disorders, including obesity and being overweight, has experienced a substantial surge in recent years, with projections indicating a continued rise in the forthcoming decades. Urbanization, population expansion, aging demographics, and the widespread adoption of Western lifestyle norms stand as the primary drivers behind the escalating occurrence of metabolic irregularities. Unquestionably, there exists a legitimate apprehension regarding the global public health trajectory moving forward. In accordance with the World Health Organization (WHO), obesity is identified by a body mass index (BMI) equal to or exceeding 30, while being overweight is characterized by a BMI equal to or surpassing 25 [1,2]. The global trends report a 50% and 80% increase in overweight and obesity incidence, respectively, in adult individuals (>20 years old) from 1980 to 2015 [3]. In 2016, the World Health Organization (WHO) reported that more than 1.9 billion adults aged 18 and above were grappling with excess weight, with over 650 million among them classified as obese [4]. In fact, by 2030, it is predicted that 1.35 billion adults will be overweight, and 573 million individuals will be obese. However, if recent trends continue, the numbers will be higher [5]. The World Obesity Federation has recently predicted that by 2035, an increase of up to 24% will occur in the prevalence of obesity, expecting nearly 2 billion individuals with metabolic disorders [6]. These trends illustrate the globalization of the Western lifestyle, where obesity/being overweight is a multifactorial disease that can be described as a dysregulation of the overall energy homeostasis of the body [7].

This metabolic disorder can emerge due to shifts in lifestyle patterns. These changes encompass fluctuations in physical activity levels, initially marked by a decline and, subsequently, a surge in sedentary behaviors. Additionally, alterations in dietary practices play a pivotal role, exemplified by an escalated consumption of high-calorie diets or overindulgence. These dietary choices, often more affordable and congruent with contemporary living, contribute to the prevailing scenario. However, recent insights have underscored that the genesis of obesity is multifaceted. Beyond lifestyle influences, genetic predisposition, environmental pollutants, hormonal dynamics, pharmaceutical interactions, endocrine disruptors, and factors intricately linked to metabolism have surfaced as intertwined contributors to the onset of obesity [8,9,10]. In fact, monogenic obesity describes a disorder promoted by single-gene mutations, usually in genes associated with endocrine regulation, that results in an obese phenotype [11]. In parallel, epigenetic markers like DNA methylation and histone modifications exert their influence on genes intertwined with growth and metabolic processes. Studies have documented instances where irregularities in the imprinting process of metabolic genes disturb their typical patterns of expression. As a result, this disruption sets off metabolic imbalances, ultimately contributing to conditions such as obesity [12,13,14]. While our understanding of the precise ramifications of these epigenetic alterations on the progression of metabolic disorders remains in its primary stages, a palpable unease has emerged regarding the metabolic well-being of successive generations. Additionally, obesity/being overweight is associated with the development of several other comorbidities, such as type-2 diabetes (T2D), cardiovascular disease, hypertension, infertility, endocrine disruptors, and cancer [15,16,17]. It is suggested that obesity is responsible for approximately 20% of all cancer cases and the cause of death of approximately 20% of women and 14% of men [18]. In fact, obesity has surpassed smoking as the most widespread high-risk for carcinogenesis in the United States of America [19].

Excess weight and obesity play a pivotal role in fostering tumor development through multiple molecular pathways. The state of being overweight or obese triggers hormonal dysregulation, culminating in elevated levels of circulating leptin and estrogens. This hormonal imbalance subsequently fuels cell proliferation across various carcinoma types (breast [20,21], colon [22], pancreas [23], and others), contributing to the intricate network that promotes carcinogenesis. Meanwhile, chronic inflammation associated with obesity leads to the overexpression of inflammatory adipokines by activated macrophages, such as Interleukin-6 (IL-6) and Tumor Necrosis Factor-Alpha (TNF-α), which are both correlated with cell proliferation and carcinoma migration [24]. These alterations can also help cancerogenic cells to avoid death. Leptin and IL-6 stimuli are known to promote the expression of pro-survival factors such as the B-cell lymphoma-2 (BCL-2) in cancer cell lines [25,26,27]. Leptin was also found to promote telomerase activity [28]. The reactivation of telomerase stands as a defining trait of cancer cells, conferring upon them an unrestricted capacity for proliferation. In sum, obesity elevates the susceptibility to carcinogenesis by fostering unbounded cell proliferation and fortifying resistance against cell death mechanisms. Nonetheless, we are merely scratching the surface in unravelling these intricate mechanisms.

Among a myriad of other coexisting conditions, men afflicted by obesity exhibit an elevated susceptibility to the onset of prostate cancer (PCa) [29]. PCa stands as the second most prevalent malignancy and ranks as the fifth primary contributor to male mortality. The global prevalence of PCa continues to surge, with escalating rates not only in its occurrence but also in mortality [30]. Individuals with excess weight and obesity are at a higher risk of developing PCa. In addition to the aforementioned mechanisms, the prostate is abundant in insulin receptors. Insulin resistance linked to obesity potentially wields an influence on the progression of PCa. However, substantiating this assertion necessitates further research and comprehensive studies [31]. Additionally, the hypoandrogenism found in individuals with obesity appears to be correlated with the development of androgen-independent PCa, which severely compromises the effectiveness of hormonal therapy and is associated with worse prognostics [32]. Interestingly, leptin was proposed to be a mediator of androgen-independent PCa proliferation [33,34]. On the other hand, traditional PCa diagnosis tests have a higher probability of failing in individuals with obesity. This happens due to the increased serum volume found in these individuals, which decreases the serum concentration of Prostate-specific antigen (PSA), leading to a false negative test. Meanwhile, the enlargement of the prostate may affect the digital rectal examination (DRE), leading to a faulty diagnosis [35]. Overall, the increased risk for aggressive PCa development and later diagnosis is reflected in the high mortality rate found for individuals with obesity. In this review, we delve into the existing body of literature concerning the mechanisms through which obesity disrupts prostate homeostasis and instigates the development of PCa. Moreover, we explore the current literature concerning the potential link between the transgenerational inheritance of obesity and of PCa. 

## 2. Deciphering How Obesity Fuels Carcinogenesis—A Role for Inflammation, Hormonal Dysregulation, and Oxidative Stress

**Figure 1:**
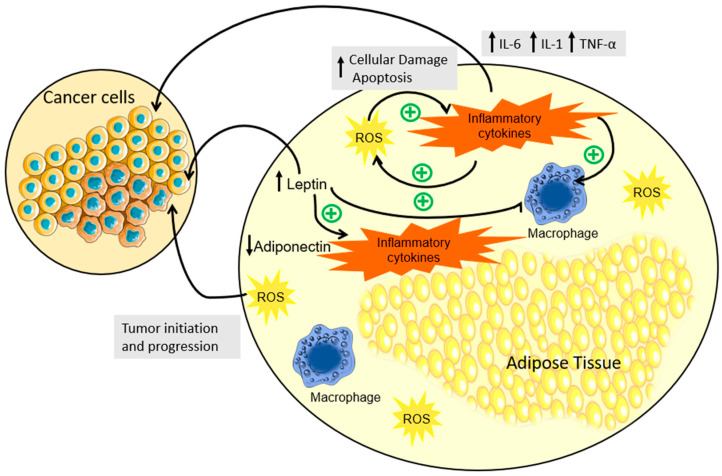
Schematic representation of inflammation, hormonal dysregulation, and OS in the adipose tissue due to obesity. In individuals with obesity, a notable expansion of adipose tissue triggers an aberrant production and secretion of cytokines, accompanied by the disruption of adipokine regulation. This cascade instigates a series of interconnected events: cytokines foster heightened ROS production, inciting apoptosis, which then exacerbates cytokine release, perpetuating a self-perpetuating cycle. This cytokine orchestration not only contributes to the perpetuation of low-grade chronic inflammation but also significantly augments the landscape for tumor development. Concurrently, elevated leptin levels in obesity correlate with heightened inflammatory cytokine levels, fostering an environment conducive to both the initiation and progression of tumors. In contrast, the diminished presence of adiponectin compounds the scenario, offering a conducive milieu for tumor development. In summary, the complex interplay between obesity, cytokine dynamics, and adipokine regulation unveils a multifaceted process that intricately contributes to chronic inflammation and the initiation and advancement of tumorigenesis. (↓) downregulation; (↑) upregulation; (+) promotion.

Obesity, as defined by the WHO, entails an abnormal and excessive buildup of fat. This atypical accumulation of fat profoundly affects numerous physiological processes, with the undeniable focal point being the extensive impact on adipose tissue. To start, excessive fat accumulation induces adipocyte hypertrophy and hyperplasia [36,37]. Hyperplasia manifests as a rise in the adipocyte count, while hypertrophy facilitates substantial fat accumulation. Nevertheless, as adipocytes progressively enlarge, blood supply diminishes, precipitating a state of hypoxia [38,39]. Hypoxia can be a stimulatory cause for necrosis and macrophage infiltration into the adipose tissue, leading to an overproduction of pro-inflammatory factors, like inflammatory chemokines (Figure 1). This results in localized inflammation, which evolves into an overall systemic inflammation associated with the development of obesity-related comorbidities [40]. The progressive enlargement of adipocytes eventually results in their rupture and consequent death. This process leads to low-grade chronic inflammation, promoted by the abnormal production of cytokines by the fat-saturated and necrotic adipocytes, which promotes the infiltration and activation of macrophages into the adipose tissue (Figure 1) [41,42]. IL-6 and TNF-α are pro-inflammatory cytokines associated with white adipose tissue expansion and saturation [41,43]. Moreover, IL-6 and TNF are also known to be tumor-promoting cytokines, with important roles in tumor initiation and progression [24,44]. In fact, before tumor initiation, inflammation can promote a favorable microenvironment in which cancer cells can thrive, stimulating cell proliferation and angiogenesis [41]. For example, the TNF-α stimuli promote the development of tissue architecture needed for tumor progression, while promoting the expression of other cytokines and factors leading to increased tumor growth and survival [45,46]. While the intricate molecular pathways governed by TNF-α remain partially elucidated, targeting TNF-α inhibition emerges as a promising avenue in cancer therapy [47]. Additionally, the activation of the innate response by the damaged adipocytes also promotes a state of oxidative stress (OS) [48]. Pro-inflammatory cytokines produced by the damaged adipocytes, such as TNF-α, IL-6, and Interleukin-1 (IL-1), promote the production of reactive oxygen species (ROS) and reactive nitrogen species by the cells of the innate immune response, increasing lipid peroxidation levels (Figure 1). Furthermore, the production of ROS at sites of inflammation intensifies cellular damage, triggering apoptosis. This, in turn, augments the release of pro-inflammatory cytokines, instigating a self-perpetuating cycle of inflammation [48,49,50]. The protein, lipid, and DNA damage induced by OS is also associated with neoplastic activation and aberrant cellular proliferation [51,52,53]. Concurrently, the oxidation of DNA bases promotes the occurrence of mutations and altered DNA methylation patterns, which can promote the abnormal expression of oncogenes [54]. This underscores inflammation as a critical hallmark of cancer, which is further potentiated by obesity. Consequently, it becomes a strategic focal point for combatting the disease.

In addition to inflammation, obesity is known to promote severe hormonal dysregulation. In fact, the adipose tissue responds to both metabolic and endocrine cues [55]. Adipokines, released by adipocytes or macrophages infiltrated in the adipose tissue, have their profile modified depending on the adipose tissue changes and their release results in metabolic alterations [56,57]. Adipokines induce low-grade chronic inflammation, insulin resistance, and the development of obesity-related diseases. Moreover, adipokines have pro- and anti-inflammatory properties [58,59]. Leptin, an adipokine and growth factor, is known to promote satiety, reduce food intake, and increase metabolic rate, preventing lipid accumulation [60]. Leptin is a powerful monocyte/macrophage chemoattractant, promoting their migration [61], and through the stimulation of macrophage activation, leptin promotes the expression of inflammatory cytokines, such as TNF-α and IL-6 (Figure 1) [62,63]. Heightened leptin levels identified in individuals with obesity are posited to constitute a component of the chronic inflammation mechanism observed in this population, as previously discussed. Additionally, leptin is a growth factor and a direct physiological agent in cancer growth. Studies have reported the role of leptin in cell proliferation, suppression of apoptosis, and tumor cell development [64,65]. Furthermore, leptin has been shown to induce telomerase activity in cancer, promoting cell proliferation, and expressing survival factors, leading to chemotherapeutic resistance (Figure 1) [66]. The actions of leptin are mediated by the transmembrane leptin receptor (ObR). When leptin binds to ObR, it induces the activation of several pathways involved in cell proliferation, survival, and invasion [67,68]. Consequently, studies underscore the significance of leptin as a potential biomarker for assessing cancer risk, particularly in individuals who are overweight or obese [69,70]. Adiponectin is another important adipokine and in contrast to leptin, its expression levels are decreased in individuals with obesity (Figure 1) [71,72]. Adiponectin is responsible for the regulation of insulin sensitivity, glucose metabolism, and fatty acid breakdown [73,74,75]. Adiponectin affects several pathways, having a crucial role in the activation of adenosine monophosphate-activated protein kinase (AMPK). Through the mammalian target of rapamycin (mTOR), AMPK interferes with cellular growth signaling and inhibits carcinogenesis promotion, tumor cell adhesion, and migration (Figure 1) [76]. TNF-α, increased in subjects with obesity, can inhibit adiponectin, which could also explain the lower expression of adiponectin found in these individuals [77]. Furthermore, OS in correlation with fat accumulation was inversely linked to plasma adiponectin levels, with evidence showing that OS downregulates the expression of adiponectin [78]. Thus, the diminished adiponectin levels observed in individuals with obesity pose a tangible risk, fostering an environment conducive to uncontrolled cell proliferation and thereby cancer progression [79,80]. 

**Figure 2:**
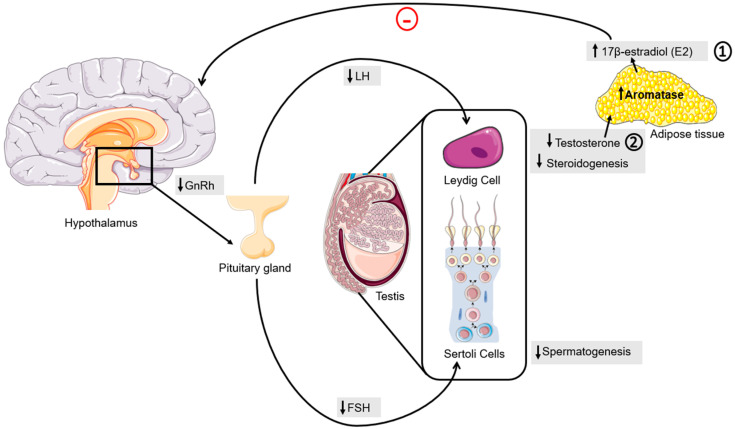
Hormonal dysregulation within the male reproductive system of individuals dealing with obesity. Two mechanisms can result in an impaired hormonal profile due to obesity. The first one corresponds to the conversion of testosterone into 17β-estradiol in the adipose tissue due to higher aromatase activity. This creates a negative loop in the HPG axis, decreasing neurohormones expression. The second one is the decrease in testosterone serum levels due to lack of neurohormones expression, decreasing steroidogenesis stimulation by the Leydig cells. (↓) downregulation; (↑) upregulation; (-) inhibition.

Regardless, the hormonal dysregulation promoted by obesity goes beyond the adipose tissue. The Hypothalamic-pituitary-gonadal (HPG) axis, the main endocrine regulator of the reproductive system, is also severely affected. The axis starts with the secretion of the Gonadotropin-Releasing Hormone (GnRH) by the hypothalamus. In the pituitary, GnRH promotes the secretion of the follicle-stimulating hormone (FSH) and luteinizing hormone (LH). Both neurohormones act on the male gonads, the testes. LH acts on Leydig cells, upregulating steroidogenesis and stimulating testosterone secretion. FSH acts on Sertoli cells, promoting spermatogenesis, while stimulating the synthesis of inhibin B, which is a growth-like factor [64,81] which feeds the negative loop that inhibits the secretion of FSH by the pituitary and stimulates the secretion of testosterone in Leydig cells [64,81,82]. Men with obesity present an impaired reproductive hormonal profile, with lower serum levels of the sex hormone-binding globulin (SHBG) and testosterone (Figure 2) [83]. This can occur through two main mechanisms. The first revolves around the conversion of testosterone into 17β-estradiol (E2) within the adipose tissue. The hypertrophy and hyperplasia of adipose tissue frequently observed in individuals with obesity subsequently trigger heightened levels of aromatase expression and activity. Consequently, individuals grappling with obesity experience elevated rates of testosterone conversion into E2. As E2 operates as an estrogen, it can perpetuate a negative loop within the HPG axis. By acting on the pituitary gland, it contributes to the reduction in neurohormone expression [84]. The second mechanism by which obesity impairs testosterone serum levels arises from the inadequate expression of neurohormones by the pituitary gland. This deficiency leads to a diminished stimulation of steroidogenesis, ultimately resulting in decreased testosterone synthesis (Figure 2) [64,85]. Meanwhile, the chronic state of inflammation found in individuals with obesity also contributes to the decreased levels of serum testosterone [86]. 

To conclude, excess weight and obesity can promote an ideal microenvironment for the development of tumors. This aligns with recent statistics indicating that obesity has now surpassed smoking as the most prevalent high-risk factor for carcinogenesis in the United States of America [19]. In the upcoming section of this study, our attention will be directed towards exploring the contribution of excess weight and obesity to the progression of PCa.

## 3. Obesity Is an Amplifier Factor for PCa Development

The prostate, situated in the lower pelvis just below the urinary bladder, is a composite organ composed of glandular and muscular elements. As an integral part of the male reproductive system, it functions as an accessory gland [87]. This gland is divided into different histologic zones: the peripheral, transition, and central zones. The peripheral zone is the region of origin of most prostate cancers, consisting of 70% tissue in normal cancers. The transition zone consists of about 5% of the prostate and is enlarged by benign prostatic hyperplasia (BPH), which is a common benign proliferation. Nevertheless, tumors that develop in the transition zone tend to stay in this area. The central zone, consisting of around 25%, is not a site of origin of any disease but still can be affected by cancer [88,89]. The growth and differentiation of the prostate is mainly under androgen control but is also dependent on other steroid and peptide hormones. Both genetic and environmental factors can lead to the development of cancer cells through various molecular mechanisms. Initiation of prostate carcinoma often involves the accumulation of cancerous cells, with changes in cell compartments. There are two possibilities for the cellular origin of PCa [90]. The first one is the transformation of final differentiated luminal cells, leading to partial dedifferentiation and immortality status. The other possibility consists of the oncogenic modification in prostate stem cells, believed to be located among basal cells [91]. These cells will originate proliferative cells with a luminal phenotype, consisting of the tumor [92]. In older individuals, BPH can occur due to prostate changes. BPH typical characteristics include basal layer expansion and hyperproliferation of the stromal. On the other hand, prostatic intraepithelial neoplasia (PIN) is an in situ carcinoma, considered a precursor to invasive prostate carcinoma. The transition from PIN lesions to adenocarcinomas involves various histological changes in the invasive epithelial cells with a cytokeratin profile, such as excessive branching morphogenesis, basal cell layer lost, cytologic atypia with nuclei, and nucleoli expansion [91,93]. In early PCa stages, an important change occurring is the formation of proliferative inflammatory atrophy regions of epithelial cells that are related to inflammation in the stromal compartment. These regions present a higher cell proliferation and are proposed as a precursor to PIN [94,95].

**Figure 3:**
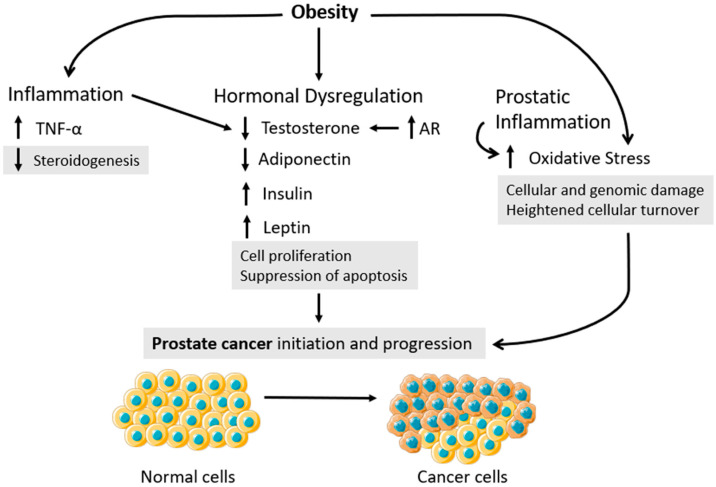
Schematic representation of obesity-related factors inflammation, hormonal dysregulation, and OS in the initiation and progression of PCa. In individuals with obesity, lower levels of testosterone are observed, with the chronic state of inflammation contributing to this phenomenon. In fact, higher levels of TNF-α can inhibit steroidogenesis. Concurrently, lower levels of testosterone are needed for the initiation and progression of PCa, along with upregulation of AR expression. Moreover, in individuals with obesity, lower levels of adiponectin are observed, as well as higher levels of insulin and leptin. This hormonal dysregulation results in the development of PCa, with leptin participating in the increase in cell proliferation and suppression of apoptosis. In turn, obesity and prostatic inflammation promote OS, leading to PCa progression, as well as cellular and genomic damage, while increasing cellular turnover. Overall, all these obesity-related factors result in the initiation and progression of PCa. (↓) downregulation; (↑) upregulation.

Hence, the prostate is under androgen control and, thus, the Androgen Receptor (AR) presents an important physiological role in its normal function. More notably, AR is crucial for PCa development and proliferation. In normal tissue, the AR is responsible for maintaining the differentiation of endogenous stromal cells, inhibiting organ growth. As previously mentioned, the majority of PCa cases manifest as adenocarcinomas, stemming from the epithelial cells. The prostate epithelium comprises two key cell types—basal and luminal—which both express AR. For decades, the prevailing theory was that elevated testosterone levels could stimulate the proliferation of PCa. Consequently, various therapeutic approaches targeting PCa, including castration and Androgen-Deprivation Therapy (ADT), have been centered around the primary goal of reducing androgen levels [96]. Additionally, lower androgen levels have been associated with decreased PCa progression, but recent evidence suggests that the correlation between PCa and androgen levels is not as simple as initially thought. To begin with, there was no correlation between PCa proliferation and testosterone levels. Witao and colleagues reported that physiological levels of testosterone could inhibit the proliferation of PCa cells in vitro, while suggesting that lower testosterone levels were essential for PCa initiation [97,98]. Meanwhile, supraphysiological testosterone levels were found to decrease the proliferation of PCa, as suggested by several authors [99,100,101]. Nowadays, the influence of testosterone on PCa development is recognized as having a dual nature. Lower testosterone levels are requisite for the initiation and progression of tumors (Figure 3), while supraphysiological levels hinder these processes. The impact of testosterone on PCa is intricately regulated by AR. The role of AR expression levels is pivotal in mediating the effects of testosterone on PCa. The therapeutic efficacy of supraphysiological testosterone levels in treating PCa can be attributed to the elevated AR expression observed in PCa cases. This upregulation of AR expression in PCa is suggested to be a compensatory mechanism aimed at counterbalancing the reduced levels of testosterone necessary for initiating tumor growth (Figure 3) [102].

Obesity is responsible for approximately 20% of cancer cases, promoting carcinogenesis in end-organs affected by adipose inflammation, OS, and hormonal dysregulation (Figure 3) [18,103]. Individuals with obesity present lower serum testosterone levels [104], with the chronic state of inflammation contributing to this phenomenon [86]. In fact, TNF-α can inhibit Leydig cells’ steroidogenesis (Figure 3) by decreasing the expression levels of the steroidogenic acute regulatory protein (StAR), responsible for the transport of cholesterol from the outer to the inner mitochondrial membrane, which is an essential step of steroid hormone synthesis [105]. TNF-α also appears to induce the expression and activation of the dosage-sensitive sex reversal adrenal hypoplasia congenital critical region on the X chromosome, gene 1 (DAX-1), which is a protein known to be involved in the inhibition of steroidogenesis by Leydig cells [106]. As a natural consequence, it is anticipated that men grappling with obesity would exhibit diminished fertility potential in comparison to their normal-weight counterparts, which is a notion substantiated by numerous studies [64,81]. The epigenetic alterations observed in the sperm DNA of men with obesity, further compounded by an elevated risk of disease, metabolic irregularities, and diverse forms of cancer in their offspring, underscore the intricate interplay between paternal health status and its lasting impact on the generations to come [64]. In essence, these findings emphasize the far-reaching implications of obesity not only on an individual’s health but also on the health trajectories of future generations. As mentioned earlier, lower levels of testosterone are important for PCa initiation and proliferation. Also, lower testosterone levels are associated with more aggressive phenotypes of PCa, largely due to the ineffectiveness of traditional treatments that aim to decrease androgen levels [35]. Unsurprisingly, a meta-analysis study has associated obesity with a higher risk of developing aggressive phenotypes of PCa, which are resistant to traditional treatment therapies [107]. Nevertheless, excess weight does not seem to be a key risk factor for PCa, unless it is associated with altered testosterone or other androgen levels [55].

Obesity is also associated with increased levels of E2. It has been suggested that the administration of E2 could be beneficial for the treatment of PCa. Steg A and Benoit G reported that the administration of percutaneous E2 to patients with untreated PCa promoted a significant decrease in serum testosterone levels while lowering LH and FSH levels as well, which the authors concluded to be beneficial for the treatment of PCa [108]. Montgomery B and colleagues used a castration-resistant PCa orchiectomized mice model to test the efficacy of E2 on the treatment of PCa. The authors reported that treatment with E2 was able to significantly suppress tumor testosterone and dihydrotestosterone levels while inhibiting tumor growth through the modulation of estrogen receptors [109]. The molecular mechanisms by which E2 exerts its effects on PCa are yet to be unveiled. Nevertheless, it has been suggested that the impact of E2 on PCa is primarily governed by the interplay between ER-α and ER-β receptors. It has come to light that PCa cell lines exhibiting a higher ratio of ER-α to ER-β tend to display heightened aggressiveness, with E2 seemingly exacerbating OS. Conversely, within PCa cell lines characterized by a lower ER-α/ER-β ratio, E2 encourages the upregulation of uncoupling proteins (UCPs) and other antioxidant enzymes [110]. As far as we know, no correlation has been established between obesity and high levels of serum E2 with the development, progression, or aggressiveness of PCa [111]. Nonetheless, a more cohesive correlation has been established between the increased expression of adipokines, in particular leptin, in individuals with obesity and the development and progression of PCa (Figure 3). Obesity can particularly affect the prostate’s physiology since prostate epithelial cells express adipokine receptors, making it more susceptible to the hormonal alterations imposed by this condition [112,113]. As previously mentioned, adipokines are known to be released during fat mass expansion occurring in obesity and cause metabolism alterations, inducing the development of obesity-related disease. Studies have indicated a potential role of adipokines and obesity in cancer progression, suggesting an important contribution to disease progression and risk, as reviewed by Booth and colleagues [114]. Leptin is associated with increased inflammatory cytokines [115], macrophage activation, and ROS production [116,117]. This adipokine acts in GABA (γ-aminobutyric acid)-ergic neurons in order to prevent the effects caused by obesity [118]. Leptin might be a predictor of advanced PCa in patients with obesity, hence, higher leptin is associated with PCa risk [119]. Concurrently in PCa, leptin was shown to increase cell proliferation and suppression of apoptosis, thereby enhancing tumor growth (Figure 3) [120]. In PCa cell lines, DU145 and PC-3, leptin-induced expression of the vascular growth factor (VEGF), transforming growth factor-β1 (TGF-β1), and basic fibroblast growth factor (bFGF) resulted in the stimulation of cell survival pathways, proliferation, and angiogenesis [121]. Furthermore, leptin has been shown to upregulate aromatase expression [122]. On the other hand, adiponectin helps to attenuate liver inflammation and fibrosis through AMPK activation and peroxisome proliferator-activated receptor-α pathways, increase free fatty acid oxidation, and reduce inflammatory cytokine suppression, as well as anti-inflammatory cytokine induction [123,124]. Most studies found lower levels of adiponectin in PCa, as reviewed by Angel and colleagues (Figure 3) [125]. Dysregulated adiponectin may contribute to tumor initiation or progression due to its anti-inflammatory and antiangiogenic properties [126]. The clinical association between PCa and obesity might also be explained by the levels of adiponectin in individuals with obesity since adiponectin can inhibit OS in PCa. The underlying mechanism involves the induction of anti-oxidative enzymatic defense mechanisms [127].

Insulin-like Growth Factors (IGFs), signaling molecules synthesized by a wide array of body tissues, play a pivotal role in the landscape of carcinogenesis. Elevated IGF expression is linked to the stimulation of cell cycle advancement and the impediment of apoptosis. This influence is enacted through direct and indirect interactions with oncogenic pathways [128,129]. Obesity causes damages that can trigger an escalation in insulin resistance, prompting an elevated requirement for insulin to maintain optimal glucose levels [130]. This phenomenon takes place when there is a decrease in glucose transportation stimulated by insulin and metabolic processes within adipocytes. Such alterations can be attributed, in part, to compromised insulin signaling in adipocytes, leading to the downregulation of GLUT4, which is a pivotal glucose transporter responsive to insulin [131]. Both insulin and IGF play pivotal roles in instigating various mechanisms that foster tumor growth, encompassing cellular proliferation and differentiation, angiogenesis, and anti-apoptosis [132,133]. Moreover, there is growing evidence linking insulin to the process of carcinogenesis [134,135,136] and PCa growth stimulation (Figure 3) [137]. In this study, the authors used a transplantable rat-derived cell line expressing PCa, PA-III, to test the effects of insulin and IGFs on cell proliferation. Two different IGFs were used, IGF-I and IGF-II, which are thought to be associated with cancer progression. The results revealed an increase in cells and DNA synthesis in the presence of insulin and IGFs in a dose-dependent manner. Additionally, both IGF-I and IGF-II showed a binding on PA-III cells with affinity of receptor sites [137]. In the context of PCa cell lines, a reduction in insulin and IGF-1 levels has been shown to yield diminished growth and increased apoptosis [138]. In the realm of human health, elevated insulin levels have been identified as a risk factor for both the development and recurrence of PCa (Figure 3) [139,140]. Furthermore, the convergence of abdominal obesity and heightened insulin levels has been linked to an escalated risk of PCa [139,141]. A study conducted by Hsing and colleagues unveiled a direct association between insulin resistance and an increased PCa risk, while insulin sensitivity was correlated with a diminished risk of PCa occurrence among Chinese men [31]. Cancer has been linked to insulin by different mechanisms. The first one is in cases of chronic hyperinsulinemia; hence, insulin exerts oncogenic potential through abnormal stimulation of various cellular pathways. This enhances growth factor-dependent cell proliferation and/or directly affects cell metabolism. Secondly, since obesity causes a low-grade inflammatory state, a higher production of IL-6, adiponectin, leptin, or TNF-α is present. Therefore, a transformation and progression in cancer are seen, as previously described [142]. The third one involves the suppression of SHBG production by insulin, which increases testosterone levels. Lastly, insulin increases IGF-I production in the liver, while reducing production of the insulin-like growth factor binding proteins 1 (IGFBP-1) and 2 (IGFBP-2). Even though insulin can induce direct tumor growth, its mitogenic and antiapoptotic effects also result in tumor cell growth [142,143]. The last two have already been related to PCa. 

Obesity-associated OS is proposed to be involved in the conversion of androgen-dependent PCa into androgen-resistant prostate cancer (ARPC) through the regulation of AR expression. Androgens can have pro-oxidative effects through the activation of pro-oxidative signaling pathways, such as fatty acid oxidation in the mitochondria. This pro-oxidation state is further promoted by the downregulation of ROS detoxifying enzymes and upregulation of NADPH oxidases [52,144,145]. OS has been associated with prostate hyperplasia development [53]. It is known that it significantly contributes to both the initiation and progression of PCa, exerting its influence on pivotal molecules such as DNA, transcription factors, and cell cycle regulators [53]. Moreover, the interplay of antioxidants and protective molecules against OS has been demonstrated to play a preventive role in PCa. Within the realm of antioxidant systems, a network of enzymes and enhancer elements operates to shield cells from the ravages of OS. These components encompass enzymes found in the glutathione redox system, as well as enhancer elements such as antioxidant response elements (ARE). This intricate defensive response is triggered in reaction to escalated levels of ROS [146]. For instance, within the realm of the glutathione redox system, enzymes like glutathione-s-transferases (GST) and glutathione peroxidase (GPx) take center stage. GST is instrumental in the detoxification of activated procarcinogen metabolites, influencing the cell’s sensitivity to various harmful chemicals [147]. Simultaneously, GPx functions as an electron donor, quelling peroxyl radicals and thus diminishing cellular OS, which in turn curbs the progression of PCa [148]. On another front, the activation of ARE leads to the transcription of genes responsible for enhancing antioxidant defenses [149]. 

OS can also be further intensified by prostatic inflammation (Figure 3). Prostatic inflammation is commonly linked (though not universally) with pathological infections that induce swelling of the prostate tissue, leading to painful and challenging urination as well as a general sense of discomfort. This inflammatory state within the prostate is posited to play a role in the development and advancement of PCa, serving as a factor not only in the initiation of carcinogenesis but also in causing cellular and genomic damage, while fostering heightened cellular turnover (Figure 3) [53]. Prostatic inflammation may be a risk of high-grade PCa, which is a condition further aggravated by the inflammation promoted by obesity [150]. Additionally, both adipose and prostate tissue have their DNA integrity and epigenetic regulation disturbed due to excessive adiposity [151,152].

## 4. Can Paternal Obesity Be Promoting PCa Development in the Offspring?

Epigenetic transgenerational inheritance is a highly conserved mechanism characterized by the transmission of phenotypes through generations in the absence of exposure to the original trigger in the germ cells of the developing fetus [153,154,155]. Transgenerational inheritance through the paternal line is established in two generations, except for when the F0 female is pregnant during exposure to the effect [156,157,158]. Environmental factors such as pollution, organic chemicals, metals, microbiome, diet, smoking, parental style, and cultural features can cause epigenetic modifications. Furthermore, these environmental stressors can alter epigenetic patterns, which might have an impact on biological or health-related outcomes. Histone modifications and DNA methylation are two examples of epigenetic modifications [159,160,161]. In fact, as reviewed by Suter and Aagaard-Tillery, epigenetic marks such as methylation, phosphorylation, and acetylation may explain behaviors such as fear conditioning, drug addiction, and behavioral features associated with mental illness [162]. Moreover, endocrine disruptors, a group of environmental compounds that affect normal endocrine signaling, have been implicated with epigenetic modifications, both on a multi- and transgenerational level [163]. Additionally, the gut microbiota and the molecules produced by it can play an important role in epigenetic processes [164]. Furthermore, diet can also modulate epigenetic marks such as DNA methylation/acetylation, histone modification, and even changes in miRNA expression [165].

Curiously, for several years it was thought that the spermatozoa could not carry epigenetic marking to the oocyte, and the epigenetic inheritance could only come from the maternal side. This concept has its roots in the protamination process that spermatozoa must go through to achieve chromatin condensation and protect DNA for the long voyage until it reaches the oocyte [166,167]. It was thought that protamination would erase the epigenetic marks carried on the paternal genome. Due to this, for years the epigenetic inheritance of the embryo was only attributed to the mother. Nowadays, we know that several epigenetic marks survive the protamination process, including the ones present on the 5–15% of chromatin that remains nucleosome bound [166,167,168]. In fact, gene ontology analysis has reported that several metabolic genes are present in these nucleosome-bound regions and are known to be hotspots for epigenetic modifications [169,170]. The last years have been characterized by the rise of evidence that obesity can be transgenerationally inherited by the paternal line [171]. Fullston and colleagues conducted an intriguing study involving male mice that were subjected to an HFD. These male mice were then bred with females who were lean and metabolically healthy [172]. Remarkably, the offspring—comprising both males and females—exhibited metabolic irregularities stemming from this paternal obesity. The initial impact was observed in terms of increased adiposity in the offspring, and intriguingly, this effect was exclusive to the daughters. Nevertheless, both male and female descendants exhibited compromised glucose tolerance and reduced insulin sensitivity. Astonishingly, even in the subsequent F2 generation, these metabolic aberrations persisted when descendants—both sons and daughters—were mated with partners who were lean and metabolically healthy [172]. Notably, the researchers delved further into the mechanisms at play. They found that the content of small non-coding RNAs (sncRNAs) within the spermatozoa of the F0 generation was altered, alongside discernible changes in DNA methylation patterns [172]. Crisóstomo and colleagues have also demonstrated that the consequences of an HFD until adulthood can also impose several alterations on testis metabolism that are not reverted by a diet switch [173]. The spermatozoa derived from these animals exhibited notable changes in the expression of sncRNAs. Intriguingly, these alterations were also discernible in the spermatozoa of the offspring stemming from animals exposed to this transitional diet [173]. These sncRNAs, which were formerly considered to lack function, have now emerged as pivotal players in the regulation of various processes, including embryo development. Chen and his team revealed a significant shift in the expression of a specific subset of sncRNAs—transfer-RNA derived small RNA (tsRNA), primarily originating from the 5′ halves—in spermatozoa from mice with obesity subjected to an HFD [174]. In a follow-up, the researchers extracted this pool of tsRNAs from these spermatozoa and introduced them into zygotes derived from normal, metabolically healthy parents. While the resulting pups did not exhibit remarkable changes in body weight compared to those raised on standard diets, they did, however, display glucose and insulin intolerance—akin to the offspring of males fed HFDs [174]. In sum, this research has illuminated the multifaceted interplay of epigenetic mechanisms, sncRNAs, and obesity in shaping the health destinies of subsequent generations. It serves as a clarion call for further investigation into the complex landscape of transgenerational health impacts, shedding light on the enduring legacy of parental choices and metabolic status in the realm of health and disease.

Metabolic disorders can be the result of a single gene mutation or a complex interaction between the environment and genetic factors. Studies have reported the association between epigenetic mechanisms, the development of obesity, and even diet modifications. Factors like hypoxia and OS, intricately linked with obesity, have also been implicated with epigenetic modifications [153,156,158]. Obesity, characterized as a metabolic disorder, is intricately linked to a spectrum of comorbidities. Notably, insulin resistance, T2D mellitus, and respiratory and cardiovascular ailments are among the prominent associations. In the cardiovascular domain, numerous conditions exhibit heightened risks in individuals grappling with obesity. This encompasses hypertension, dyslipidemia, disorders in lipoprotein metabolism, and the emergence of diabetic cardiomyopathy—all of which often coincide with diabetes [175,176]. Recent studies have consistently highlighted the link between obesity, cardiovascular diseases, and the transmission of specific genetic markers, underscored by the intricate workings of epigenetic mechanisms. Investigations have demonstrated a robust connection between maternal obesity during pregnancy and subsequent metabolic tissue modifications in offspring, alongside consequential epigenetic changes. Ultimately, these factors contribute to the emergence of cardiovascular disorders intricately linked with obesity [177,178].

On the other hand, cancer initiation and development are known to be also promoted by genetic causes [179]. Cancer is characterized by complex genome alterations, implicating the association of the disease with epigenetics and possibly its contribution to the development or promotion of carcinogenesis. Moreover, modifications in methylation patterns might be related to the promotion of cancer cell growth or even the activation of tumor suppressors [155]. When it comes to PCa, first-degree relatives’ subjects have a two- to three-fold higher risk of developing the disease, and an increased risk is presented in a patient with several relatives affected [180]. As of now, our understanding of the transgenerational transmission of PCa remains limited, largely due to the intricate nature of the inherited genetic predisposition to this condition. While the specifics of inherited susceptibility to PCa are not fully elucidated, certain sites seem to exhibit heightened vulnerability and a robust genetic influence is notably evident within familial contexts. Nonetheless, despite this intricate genetic component, it does not appear to interfere with the process of genetic transmission [181]. Furthermore, PCa appears to be one of those cancers explained by autosomal dominant inheritance, and studies have associated PCa not only with genetic factors but also with environmental ones, like excess adiposity. Nevertheless, research with families shows that hereditary PCa occurs at a younger age, in comparison to sporadic PCa, and it has a higher heritability in men than any other cancer [182,183]. Additionally, even though several works have explored the role of obesity in the development of PCa, as it was reviewed in this work, to our knowledge, no work has been performed regarding the impact of obesity and the inheritance of PCa to future generations. With the rise of the obesity pandemic, it is important to understand how obesity can modulate the transgenerational inheritance of other disorders, identify at-risk individuals, and propose a better diagnosis and treatment. The health of future generations is at risk. 

### 4.1. Is It Possible to Determine a Biomarker for the Inheritance of PCa (And Obesity)?

The most classical PCa biomarker used for the diagnosis is PSA, a member of the kallikrein family. Its higher levels are useful for the detection of PCa since an increase in PSA is often indicative of PCa. This biomarker is secreted by prostate epithelial cells, being present in the serum of patients with prostate disease, such as PCa, BPH, and acute prostatitis [184]. PSA detection has a low specificity and sensitivity for PCa since its levels can be affected by another prostatic disease, age, and depends on race [185]. On the other hand, a lower level of PSA has been associated with individuals with obesity, which can lead to a delay in the diagnosis of PCa. This association can be explained by hemodilution, a condition characterized by the increased blood volume found in individuals with obesity, which causes the dilution of tumor markers like PSA [186]. The diminished levels of testosterone typically observed in these individuals are concurrently linked to reduced PSA production. Nonetheless, the precise underlying mechanism that ties together obesity and the decline in PSA levels remains enigmatic [187,188,189,190]. Since obesity imposes an obstacle to cancer screening and early diagnosis, individuals with obesity are normally diagnosed with advanced stages of the disease. Taking this into consideration, it is imperative to find new biomarkers for PCa diagnosis in individuals with obesity [191,192]. 

#### 4.1.1. Androgen Receptor

As discussed, AR is present in all phases of PCa progression but is not singly responsible for the growth of PCa cells. AR is required for PCa initiation since the stimulation of low levels of testosterone is an important step in this process. Additionally, while AR is associated with cell differentiation in the normal prostate tissue, in PCa it promotes proliferation [193,194]. Elevated expression levels of AR have been correlated with instances of treatment failure in androgen ablation therapy. This is due to the phenomenon wherein even in the presence of low testosterone levels, tumor progression can still be promoted [195,196]. Furthermore, it is worth noting that antiandrogen therapies can induce DNA damage and heightened radiosensitivity. This outcome is linked to the engagement of AR stimulation in specific DNA repair processes [197]. 

Obesity is known to alter both the volume and composition of the adipose tissue, which in turn will influence lipid availability and utilization [198]. Men with obesity have more aggressive disease and a higher recurrence rate following surgery [35]. Cancer cells are known to have high survival requirements such as the need for increased lipids, which are used for membrane production, energy, and intracellular signaling. Cancer cells can acquire lipids by the overexpression of enzymes for de novo lipid synthesis or by the uptake of exogenous lipids from circulation. PCa cells tend to utilize fatty acids as energetic substrates and lipids utilized by these cells can drive proliferation and invasiveness [199]. Androgens and AR are known to regulate lipid metabolism, an important link to carcinogenesis. AR mediates lipid biosynthesis, a highly conserved process able to influence the lipid profile of prostate cells. In more advanced and aggressive cases of PCa, intense lipid signals and droplets are present. When lipid biosynthesis is reactivated via AR, PCa progresses to Castration-Resistant Prostate Cancer (CRPC). In the context of ADT, the augmentation of lipid biosynthesis inhibition leads to a reduction in tumor growth [199,200,201]. Regardless, through the indirect activation and enhanced expression of sterol regulatory element-binding proteins (SREBPs), androgens can stimulate de novo lipogenesis and lipid uptake. Hence, to regulate this process, a reciprocal relationship between AR and SREBP is essential [199,202]. LNCaP, an established human prostate carcinoma cell line, has been used in the study of PCa progression, during different stages and in response to several therapies, specifically involving AR signaling, since LNCaP cells present the unique ability to express AR [203]. A noticeable effect of androgens on LNCaP cells is the accumulation of neutral lipids. Since these lipids are de novo synthesized, LNCaP cells are thought to express all the enzymes needed for endogenous lipogenesis. Some of these enzymes have their expression and/or activity affected by androgens. AR mediates androgens, which stimulate the expression and activity of the fatty acid synthase (FASN). FASN stimulation represents part of the mechanism by which androgens induce neutral lipid accumulation [204]. In fact, abnormal FASN expression has been associated with PCa progression via lipid metabolism dysregulation [205]. A study by Huang and colleagues demonstrated that SREBP-1 can promote PCa growth and progression via AR/lipogenesis axis. The authors concluded that SREBP-1 induced the expression of AR and FASN, which resulted in fatty acid induction and lipid droplets in PCa cells. Moreover, SREBP-1 stimulated ROS levels, a mechanism known to contribute to carcinogenesis. Overall, SREBP-1 was shown to regulate PCa proliferation and development in LNCaP cells [206]. Taking all of this into account, AR might be a link between obesity and PCa, making it a useful biomarker for individuals with obesity.

In some cases, a genetic component is associated with increased PCa risk. Researchers have been investigating the relationship between a hereditary component and the development of PCa. Common single-nucleotide polymorphisms (SNPs) have been associated with familial relative or the development of PCa risk in various populations, as reviewed by Benafif and Eeles [207]. AR dysregulation plays an important role in the onset and progression of PCa. These changes include point mutations (such as single-base substitution), AR overexpression, AR splice variants, androgen biosynthesis modifications, or AR cofactor alterations. Most of the mutations in AR variants identified in PCa tissue rarely occur within the germline. Mutated AR variants result in small changes in the AR protein. For example, the mutation AR^RT877A^ is the most frequent. This mutation consists of the substitution of an alanine for a threonine, leading to specificity loss for the agonist. Therefore, the mutated AR can be activated also by steroid hormones and lead to tumor growth [208,209,210]. Nevertheless, specific AR germline polymorphisms have been associated with an increased risk of developing PCa and therefore may have a potential transgenerational effect [193]. A groundbreaking research conducted by Hu and his team unveiled a pivotal mutation stemming from a germline substitution within the AR of three African American family members, all of whom had a documented history of early-onset PCa. This mutation has been conclusively linked to the advancement of the disease and exerts a profound influence on AR signaling. The modification in the DNA-binding affinity induced by this mutation results in altered responses to androgens, anti-androgens, and non-androgenic steroids, illuminating a previously uncharted dimension of the disease’s molecular intricacies [211]. Additionally, an article with Finnish PCa patients revealed that the R726L substitution in AR may increase PCa risk and contribute to its progression. Moreover, it contributed to familial and sporadic PCa in the Finnish population [212,213]. On the other hand, few studies have analyzed if *AR* expression can be transgenerationally affected. For example, grandmothers of F2 were exposed to a mixture of polychlorinated biphenyls during late gestation. Preliminary data reported reduced *AR* mRNA expression in the F1 males in the preoptic area and increased expression in F2 [214]. Another study exposed pregnant female rats to two different doses of Bisphenol A (1.2 µg and 2.4 µg) during the perinatal period. The 1.2 µg dose revealed a decreased *AR* expression in the testicular tissue of rats from all three generations. The 2.4 µg dose only presented a decrease in the *AR* expression in the F3 generation when compared with the control (CTRL) groups [215]. 

#### 4.1.2. Homeobox B13

**Figure 4:**
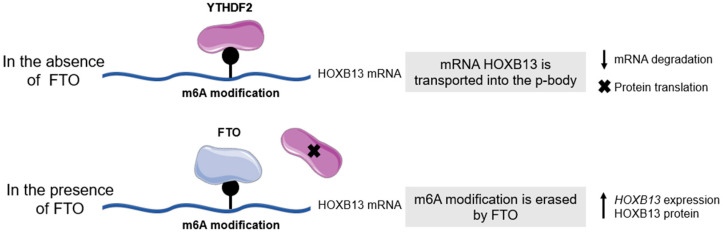
m6A modification in HOXB13 mRNA in the absence and presence of FTO. The dynamic landscape of m6A modifications within HOXB13 mRNA reveals an intriguing dichotomy that hinges on the presence or absence of FTO. In the absence of FTO, the m6A modification attracts the binding of the YTHDF2 protein. This interaction orchestrates the translocation of mRNA to the p-body, a process that expedites mRNA degradation while simultaneously impeding the translation of proteins. In a distinct scenario where FTO is a participant, its presence holds the key to a transformative change. Acting as a molecular recognition system, FTO identifies the m6A modification and initiates a catalytic cascade, ultimately leading to the demethylation of the modification. This orchestrated demethylation event serves as a trigger, ushering in an upsurge in the expression of HOXB13. This intricate regulatory mechanism highlights the pivotal role of FTO in orchestrating a finely tuned balance between mRNA degradation and the augmentation of protein synthesis within the context of HOXB13 expression. (×) absence of YTHDF2.

Localized on chromosome 17, Homeobox B13 (HOXB13) is a homeobox transcription factor with mutations being reported in 0.7% to 1.4% of PCa cases [216]. The homeobox genes encode for nuclear proteins and possess the main function of being transcription factors. This class of genes is highly conserved and crucial for correct embryonic development [217]. HOXB13 presents important roles in the regulation of the AR target gene, in the differentiation of the epithelial of the prostate gland, and in the development of the prostate and its secretory functions [218]. The interaction between HOXB13 and AR is complex. It was reported that suppression of the AR activity along with overexpression of HOXB13 has been seen in androgen-independent tumors [219,220]. HOXB13 exhibits a dual role in the regulation of prostate cell growth by effectively suppressing it through the inhibition of androgen-mediated signaling. Paradoxically, several studies have unveiled an intriguing association between elevated HOXB13 levels and the progression of tumor growth [217,221,222,223,224]. This complex relationship makes the heightened expression of *HOXB13* a potential diagnostic marker for PCa. The predominant localization of HOXB13 within the nucleus, accompanied by sporadic presence in the cytoplasm, grants it remarkable sensitivity and specificity for PCa detection [225]. The hereditary occurrence of PCa is relatively rare, accounting for merely 0.6% to 6.25% of patients [183,226]. Importantly, a significant correlation has been established between hereditary PCa and increased familial risk with aberrant *HOXB13* expression, as indicated by reference [227]. During the early stages of mouse embryogenesis, HOXB13 levels remain low and are concurrent with AR presence. Intriguingly, the knockdown of HOXB13 triggers noteworthy perturbations in pathways associated with the cell cycle, encompassing DNA replication, G1/S phase transition, and metaphase checkpoint. Notably, murine models with HOXB13 loss-of-function mutations exhibit discernible prostate abnormalities [228,229]. Concomitantly, a notable germline mutation in *HOXB13* has garnered attention due to its association with both PCa risk and hereditary PCa. Carriers of this mutation exhibit a heightened susceptibility to developing PCa [227,230]. The G84E missense mutation, identified as a founder mutation in Nordic populations, displays carrier frequencies ranging from 0.2% to 1.4%. In Western European populations, these frequencies range between 0.1% and 0.5% [231]. This mutation is situated within the evolutionarily conserved functional domain of HOXB13, suggesting its involvement in promoting PCa. Despite this, the precise underlying mechanism remains elusive. However, as suggested by others, it is plausible that the interaction between the HOXB13 domain and members of the MEIS protein family contributes to this mechanism, though further investigation is warranted [232]. On the other hand, HOXB13 activity and expression are related to lipogenesis. A study by Lu and colleagues found that HOXB13 directly suppresses lipogenic transcriptional programs by its interaction with HDAC3, a histone deacetylase. HDAC3, once activated and recruited to lipogenic enhancers, catalyzes histone deacetylation of target genes and suppresses lipogenic programs. Hence, a captivating mechanism emerges wherein HOXB13 exerts a suppressive influence on de novo lipogenesis by directly engaging with lipogenic enhancers within PCa cells [233]. Notably, this effect is achieved through its direct interaction with HDAC3, a pivotal cofactor that contributes to the intricate process of epigenome remodeling. This collaborative partnership between HOXB13 and HDAC3 underpins the orchestration of regulatory events critical to cellular function [233,234]. Furthermore, the attenuation of HOXB13 expression provides a window for the expression of pivotal lipogenic regulators, which orchestrates a cascade of events. This ultimately results in lipid accumulation within PCa cells—a hallmark of carcinogenesis—propelling heightened cell motility when examined in vitro and instigating the alarming phenomenon of tumor metastasis in vivo [233]. Intriguingly, the same investigation suggests that the expression of HOXB13 registers an upswing during the initial phases of PCa, solidifying its role as an indispensable AR cofactor that fuels the augmentation of PCa growth. However, the dynamics take a compelling twist in Metastatic Castration-resistant Prostate Cancer (mCRPC), where the levels of HOXB13 are observed to undergo a decline. This transformation in its expression profile is further mirrored by an increase in its methylation levels, underscoring a remarkable dichotomy that potentially contributes to the intricate landscape of CRPC [233]. *HOXB13* expression has also been associated with the activity of the Fat mass and Obesity gene (FTO). FTO is associated with obesity, body weight, fat mass, and BMI [235]. Despite the scarcity of comprehensive insights into its precise functional attributes, FTO stands as a genomic locus in the context of adiposity. It has been proposed to wield a substantial influence over the regulation of feeding behaviors and energy expenditure, thus positioning it as a key player in the intricate interplay governing these physiological processes [236]. Situated on chromosome 16q12.2, the FTO gene garners attention for its robust conservation across an array of vertebrate species, reinforcing its evolutionary significance as a genetic entity [237]. Unveiling its relevance in the realm of obesity, a noteworthy revelation emerges from bariatric surgery outcomes. An impressive 71.2% of individuals subjected to bariatric surgical interventions were found to harbor at least one risk allele associated with the FTO gene [238]. This intriguing finding suggests a potential modulatory role of FTO in influencing the efficacy and consequences of such surgical interventions, accentuating its potential impact on therapeutic outcomes. On top of that, FTO acts as a demethylase enzyme and has the capacity to interfere with the alternative splicing patterns of genes associated with adipogenesis. An intriguing hypothesis, conceptualized by Zhao X. and fellow researchers, establishes a compelling link between the demethylation of N6-methyladenosine (m6A) modification, the FTO gene, and the intricate process of adipogenesis. This hypothesis finds its basis in the realm of epitranscriptomic nucleotide modifications, which wield a profound influence over the destiny of mRNA molecules. These modifications, akin to methylation, endure within the nucleotide sequence, imparting them with an enduring impact on cellular processes. The focal point is the remarkable inverse correlation discovered between the expression of FTO and the levels of m6A during adipogenesis. This discovery fuels the hypothesis, suggesting that FTO’s influence on gene splicing could potentially underpin the metabolic shifts associated with obesity. This intriguing proposition points towards a multifaceted mechanism that connects these molecular players to the broader context of metabolic regulation and the development of obesity [239,240]. Through this property, FTO can erase the m6A modification from mRNA, and the mature mRNA is transported to the cytoplasm. Hence, FTO indirectly regulates the mRNA metabolism. The m6A modification is known to participate in DNA repair and important cellular processes. This modification is very frequent in the RNA of eukaryotes and a predominant internal modification in the mRNA of mammals [241,242]. Without FTO, the m6A modification is recognized by the YTHDF2 protein due to the higher binding capacity between them. Then, the protein promotes the transportation of mRNA into the p-body, accelerating mRNA degradation and inhibiting protein translation [243]. The m6A modification is located in the 3′UTR region of the HOXB13 mRNA. Therefore, if FTO is not present, the YTHDF2 protein can also bind to the HOXB13 mRNA and regulate it in the way described before. When FTO is present, its protein binds to the HOXB13 mRNA and regulates it through the m6A mechanism. FTO catalyzes the demethylation modification in the 3′UTR region of HOXB13 mRNA, erasing the m6A modification recognition with the YTHDF2 protein (Figure 4). Therefore, FTO can promote the expression of *HOXB13* and accelerate it. Furthermore, the 5′ end of HOXB13 mRNA contains two CpG islands, indicating that its expression may be regulated by DNA methylation, as previously seen [233,244,245]. 

In an article related to endometrial cancer, the relationship between FTO, HOXB13, and the m6A mechanism was studied. It was found that FTO knockdown was associated with decreased *HOXB13* expression and higher m6A modification levels [245]. On the one hand, FTO silencing promoted the peak in m6A modification, which was followed by a reduction in *HOXB13* mRNA expression. On the other hand, the silencing of the YTHDF2 expression was related to increasing *HOXB13* mRNA expression. Indeed, within the realm of endometrial cancer, a noteworthy observation emerges: the expression levels of *HOXB13* exhibit an elevation within metastatic tumors. This surge in *HOXB13* expression is intricately tied to the facilitation of tumor cell invasion and the subsequent orchestration of metastatic events when examined in vitro. Furthermore, a pivotal connection becomes apparent as *FTO* expression enters the equation. The presence of heightened *FTO* expression in endometrial cancer seems to be a driving force behind metastasis, with an even more pronounced effect catalyzed by the concurrent activation of the Wnt signaling pathway. It is worth noting that *HOXB13* expression has a well-documented association with the Wnt signaling pathway, effectively amplifying the intricate web of regulatory interactions that lead to the augmentation of tumor invasion and metastasis [245]. This interplay between HOXB13, FTO, and the Wnt signaling pathway adds a new layer of complexity to our understanding of endometrial cancer progression, illuminating potential targets for therapeutic intervention and presenting a fascinating area for further investigation. In another study analyzing the expression of *FTO*, *HOXB13*, and the m6A mechanism in gastric cancer, a positive correlation between *FTO* and *HOXB13* was detected [244]. Elevated levels of both *FTO* and *HOXB13* expression have been observed in the context of gastric cancer. Intriguingly, the inhibition of their expression initiates a cascade of effects, ultimately leading to the suppression of crucial processes in gastric cancer cell biology: proliferation, migration, and invasion. Delving deeper into the molecular intricacies, the heightened expression of *FTO* seems to wield a powerful influence over HOXB13. By engaging in the demethylation of HOXB13’s mRNA, FTO contributes to the upregulation of *HOXB13* expression. This orchestrated interplay contributes to the tumorigenic properties of gastric cancer cells. Furthermore, insights gleaned from inhibition experiments have highlighted HOXB13’s pivotal role. Inhibition of HOXB13 manifests as a powerful deterrent against cell proliferation, migration, and invasion in vitro, underscoring its significance in the malignancy’s progression [244]. Thus, augmented expression of *FTO* potentially orchestrates HOXB13’s regulation by inducing a reduction in its methylation levels. This dynamic relationship further solidifies the intricate interplay between FTO, HOXB13, and the underlying mechanisms that drive gastric cancer’s aggressive behavior. These findings not only deepen our comprehension of gastric cancer biology but also offer potential avenues for therapeutic exploration. 

Epigenetic changes, distinct from modifications to the nucleotide sequence, possess the remarkable capacity to endure across generations, shaping the genetic landscape for the descendants. One prime illustration of this phenomenon lies in the modifications of methylation patterns [246,247]. This suggests that shifts observed in the expression levels of genes like *HOXB13* or modifications within its methylation patterns can be inherited, potentially spanning multiple generations in a transgenerational transmission fashion. Dysregulation of HOXB13 could be intricately linked to heightened PCa risk and the potential for its inheritance. A compelling study led by Legoff and colleagues embarked on an exploration analyzing the prostates of mice spanning both the F1 generation, directly exposed to chlordecone, and the F3 generation, which remained unexposed. Within this investigation, discernible alterations surfaced in the prostate structures, alongside the revelation of transgenerational inheritance of its epigenetic effects. This intriguing discovery underscores the profound impact of environmental exposures on the intricate interplay between genetics and epigenetics, presenting a pathway through which hereditary PCa risk might be influenced and transgenerationally transmitted [248]. In the F1 generation, a notable decline in *HOXB13* expression was distinctly evident. Conversely, when scrutinizing the third generation (F3), a trend of reduced expression persisted, though did not achieve statistical significance. This intriguing outcome postulates that the repercussions of chlordecone exposure observed in the first generation continue to reverberate through to the third, unveiling a compelling narrative of transgenerational inheritance in the realm of HOXB13 dysregulation. The authors postulated a hypothesis that gains remarkable significance within this context. Recognizing that alterations in *HOXB13* expression have been correlated with an elevated risk of PCa, the inquiry emerges: could the exposure to chlordecone, a potential catalyst for prostate cell proliferation, be mediating its effects through the avenue of HOXB13 modification [248]? This hypothesis fuels the exploration of an intricate interplay between environmental factors, gene expression dysregulation, and the broader landscape of PCa susceptibility.

#### 4.1.3. DNA Hypermethylation

The growth and development of tumors have been related to changes in DNA methylation. Moreover, it has been reported that hypermethylation associated with specific gene promoters can lead to the silencing of tumor suppressor genes [249]. One of the most frequently observed occurrences involves the heightened methylation of cytosines positioned at the 5′ end within CpG islands situated within the regulatory regions of suppressor genes [250,251,252,253]. In renal cell carcinoma lines and primary tumors, HOXB13 methylation status is correlated with a loss of *HOXB13* expression, which is also associated with tumor grade and microvessel invasion. Concurrently, HOXB13 inactivation may play an important role in carcinogenesis and its progression [252]. In gastric cancer, lower *HOXB13* expression was associated with tumor differentiation, lymph node metastasis, and depth invasion. Furthermore, a correlation between the methylation level and gene expression could be a potential predictor of malignancy degree for this type of cancer [254]. 

PCa has been linked to genetic and epigenetic modifications, mainly DNA methylation and histone acetylation. Marks such as histone modifications, altered chromatin protein expression, or DNA methylation might be useful in PCa diagnosis [255]. Events like DNA methylation, specially hypermethylation, have been described in PCa, both in advanced and metastatic prostate tumors [256,257]. Angulo and colleagues reported that 61 genes were significantly hypermethylated in 20% of PCa tumor analyses. These patterns of hypermethylation were later associated with metastasis and a negative response to ADT [258]. Events of specific somatic alterations have a higher rate of recurrence in DNA methylation, supporting the strong selective pressure for them to occur. DNA methylation can offer insights into the origin and evolution of a tumor; hence, it is a stable event [259]. Curiously, endocrine-disrupting chemical exposure in the developing prostate can increase PCa risk [260,261]. In a study by Wang and colleagues, rats were exposed to Bisphenol A during the neonatal period. The results presented the activation of the histone methyltransferase mixed-lineage leukemia 1 (MLL1) in response to PI3K/AKT signaling. Consequently, this activation led to histone H3 lysine 4 trimethylation (H3K4me3) increase at genes associated with PCa, which in turn enhanced genes related to basal and hormone-induced gene expression in prostates at heightened PCa susceptibility [262]. A similar study exposed rats on specific postnatal days to Bisphenol A and Estradiol Benzoate. The results obtained sustained that early exposure resulted in methylation modifications in the gene-specific promoter. The authors postulated that these modifications could be used as an epigenetic biomarker for PCa recurrence [263]. Additionally, the study by Legoff and colleagues also analyzed relevant histone marks in the prostate of F1 mice directly exposed to chlordecone up to the F3 unexposed generation. In F1 mice exposed to chlordecone, H3K27me3 presented a decrease and H3K4me3 showed an increase in the prostates. However, in F3 mice, while H3K27me3 also decreased, H3K4me3 presented no changes. Curiously, higher H3K4me3 levels in mice were linked to H3K27me3 lower levels. This interplay is purposed to act as epigenetic switches, being related with increased dysregulated gene activation. Therefore, disruptions in the ratio of these marks are considered to play a role in PCa. Moreover, the authors found that the changes detected in H3K4me3 of F1 prostates were present in F1 spermatozoa, which led to the hypothesis that chlordecone exposure could induce inheritable modifications in certain regions of the epigenome. In fact, most of the changes found in F1 were preserved in F3 prostates. These results highlight that some epigenetic modifications might be transgenerationally inherited [248]. 

These hypermethylation events can occur in cancer cells and have been associated with PCa [264]. Promoter methylation is affected in Glutathione-S-transferase-P1 (GSTP1), adenomatosis polyposis coli (APC), retinoic acid receptor β (RARB), and Ras-associated domain family 1 (RASSF1) in several PCa cases, making it possible to consider these genes as PCa biomarkers [265,266,267,268]. GSTP1 hypermethylation is the most commonly identified epigenetic alteration found in PCa, which can be used as a molecular biomarker for its diagnosis due to the detection in PCa and PIN, not in normal tissues or BPH [250,269]. GSTP1 encodes GST and is involved in key cellular functions, being responsible for the protection of cells from OS and chemical attacks [250]. Furthermore, the loss of function of GSTP1 could potentially predispose normal prostatic cells to undergo DNA damage, influenced by inflammation and/or dietary intake, which can lead to carcinogenesis [270].

Obesity possesses the capacity to exert its influence on methylation patterns and gene expression. This occurs due to the profound impact that dietary and nutritional alterations can have on DNA methylation within genes crucial to metabolic processes [271,272,273]. Notably, investigations have revealed that HFDs serve as promotors of epigenetic shifts, particularly within inherited genes intricately tied to metabolic pathways [274,275]. However, the intricate linkages between the effects of obesity and the reprogramming of the epigenome remain largely shrouded in mystery. This enigma extends even further to encompass aspects of transgenerational inheritance, raising questions about the potential for these effects to cascade across generations, along with the underlying mechanisms that drive such phenomena.

As discussed earlier, obesity triggers the release of pro-inflammatory cytokines, which in turn can facilitate the elevation of DNA methyltransferase 1 (DNMT1) expression and its associated enzymatic activity. Remarkably, individuals with obesity often exhibit heightened levels of DNMT1 expression. Once DNMT1 becomes activated, it selectively engages in the methylation of the adiponectin promoter, thereby fostering a heterochromatin configuration. This molecular shift prompts a consequential reduction in gene expression, achieved through the inhibition of transcription. Consequently, the hypermethylation of the adiponectin promoter—an outcome observed in mice with obesity under the influence of HFD—inevitably culminates in the suppression of gene expression [276]. In an experimental mice model simulating basal-like breast cancer, the author tested if obesity could potentially drive epigenetic reprogramming. To address this, three distinct groups were established: the CTRL diet group, the diet-induced obesity (DIO) group, and the formerly obese (FOb) group. Notably, the FOb group initially experienced diet-induced obesity before transitioning to a CTRL diet. Upon scrutiny, it was apparent that heightened DNA methylation levels were notably present within both the DIO and FOb groups, as compared to the CTRL group. Furthermore, the outcomes seemed to suggest that altering the diet alone did not suffice to reverse the epigenetic reprogramming effects induced by obesity [277]. This finding shed light on the lasting impact of obesity on epigenetic mechanisms, even when dietary conditions are altered. 

Studies by Crisóstomo and colleagues analyzed the effects of exposure to an HFD and diet correction in a transgenerational *Mus musculus* model across three generations (F0, F1, and F2) through the division into three different diets: CTRL (standard chow), HFD (carbohydrate: 35.7%, protein: 20.5%, and fat: 36.0%), and transient diet (HFD_t_ (60 days HFD, plus 140 days standard diet)), ad libitum for 200 days post-weaning [278,279]. In the first generation (F0), HFD exposure resulted in weight gain, while diet correction was able to reverse the effects induced until adulthood and lead to weight loss. In their sons (F1), the group exposed to HFD throughout life was significantly heavier and the one exposed until adulthood presented lower fat mass, while in the grandsons (F2) no differences were found. This indicates that the father’s diet has an impact on their progeny’s body composition. Interestingly, HFD exposure in F0 displayed an increase in insulin resistance and fasting glycemia. Meanwhile, their sons showed higher glucose levels, indicating altered insulin response. In the last generation, the CTRL and HFD_t_ groups had increased fasting glycemia. However, no differences were found in F1 and F2 for fasting insulinemia. Therefore, HFD exposure throughout life induces glucose intolerance, as well as insulin resistance, and reversion of diet might prevent these effects [279]. Consequently, paternal HFD exposure results in inter- and transgenerational sperm defects. F1 displayed differences in sperm morphology; mainly, the HFD_t_ group presented an increased proportion of normal sperm. On the other hand, in F2, HFD and HFD_t_ groups, showed decreased sperm counts and viability, compared to their progenitors [278,279]. Concurrently, testicular metabolism was affected by HFD, with effects persisting for generations. For example, leucine testicular levels were increased in the HFD of F1, while in their offspring levels were decreased. A similar scenario succeeded with acetate, with low testicular levels in groups exposed to HFD throughout life or until adulthood in F0 and an overcompensation in their sons, even in grandsons. Likewise, in F0 HFD_t_ testicular glutamine levels were higher, but their grandsons presented decreased levels. In contrast, inosine testicular levels were lower in the HFD group in F0, as well as in the HFD_t_ group in F2, which might lead to a pro-inflammatory environment. Lastly, in F1, the HFD group presented increased glycine testicular levels, overcompensating for the damage induced in their progenitors, and the HFD_t_ group displayed altered leucine testicular levels, which might be linked to insulin resistance [279]. Concurrently, the alterations induced by HFD in testicular metabolism were correlated with sperm defects across three generations [278,279]. Even though no differences were found in F0, paternal HFD exposure induced alterations in the reproductive axis of F1 and F2. While the HFD_t_ group in F1 showed reduced E2 levels, the HFD group in F2 presented increased levels. Additionally, in HFD_t_ of F2, higher levels of FSH and lower levels of LH were detected [278]. Further, paternal HFD exposure disrupts the antioxidant defenses in the testis and the mitochondrial function of the offspring. This was indicated by the lower catalase activity present in the HFD group in F0, which persisted in their offspring alongside reduced mitochondrial complex I and IV in this group. Followed by the lower glutathione-disulfide reductase activity in mice fed with HFD in F0. However, in the offspring of parents exposed until adulthood, it was higher [278]. Regardless, testicular fatty acids and lipid metabolites were also affected by HFD exposure, with effects persisting for two more generations. In F2, the HFD group presented reduced polyunsaturated fatty acids levels. Additionally, the testicular content of choline was disrupted in the HFD group of F1. Grandsons of mice fed with HFD until adulthood presented increased levels of ethanolamine and lower levels of phosphocholine and phosphoethanolamine. Moreover, 3-hydroxyburate, associated with testicular insulin resistance, was highly expressed in the HFD and HFD_t_ groups in F2 [278]. Overall, HFD exposure resulted in inherited metabolic memory which caused inter- and transgenerational sperm defects and metabolic changes, further able to stimulate a pro-inflammatory environment [278,279]. 

Adiponectin, a hormone intricately linked with obesity, exhibits an intriguing inverse relationship with the disease. Notably, in recent times, significant attention has been directed toward studying adiponectin, primarily due to its recognized anti-proliferative attributes in the context of carcinogenesis. An inverse correlation is present between adiponectin levels and PCa/PCa risk. Even though little is known about the mechanisms underlying this relation, adiponectin has been suggested as a link between obesity and PCa [280,281]. A study by Tan and colleagues demonstrated that endogenous adiponectin is downregulated through promoter hypermethylation in PCa. This was associated with increased tumor proliferation and invasion [281]. As previously discussed, DNA methylation of adiponectin promoter has been observed in association with obesity. Methylation of adiponectin promoter might be a potential PCa biomarker.

Periprostatic adipose tissue (PPAT) has garnered notable attention due to its correlation with PCa, with obesity resulting in an accumulation of excess fat within PPAT. This adipose tissue is known to secrete adipokines, experience a decline in adiponectin levels, and contribute to an environment conducive to PCa growth. In this context, Cheng and colleagues undertook a significant investigation into the methylation patterns of PPAT from PCa patients who were also overweight or obese. Their findings illuminated the impact of surplus adiposity on the DNA methylation of PPAT tissue within individuals with PCa. Significantly, the majority of pathways featuring promoter hypermethylated genes were interconnected with metabolic disorders that are recognized contributors to tumor development in PCa. This compelling observation suggests that the methylation patterns altered by obesity extend their influence on the modulation of the tumor microenvironment, subsequently influencing PCa development [282]. It is important to note that promoter hypermethylation catalyzed by obesity might potentially exert far-reaching effects on PCa development, with the intriguing possibility of transgenerational transmission. Nonetheless, it is worth noting that only a limited number of studies have explored the connection between obesity-induced methylation and PCa. In the broader panorama, epigenetics occupies a pivotal role in both the initiation and progression of PCa, a notion previously established. Furthermore, the intricate interplay of nutritional factors is recognized to exert an influence on the mechanisms encompassed within this process [283].

## 5. Conclusions

Obesity, a complex metabolic disorder, intricately links to the emergence of various comorbidities, including cancer, such as PCa. This interconnection establishes obesity as a substantial risk factor in the genesis of carcinogenesis. Furthermore, the realm of epigenetic modifications, interwoven between obesity and cancer, assumes a pivotal role in the perpetual adaptation of cancer cells to evolving conditions, thereby facilitating their proliferation and viability. The hallmark characteristics of obesity, encompassing hormonal dysregulation, OS, and persistent low-grade inflammation, converge in tandem with inflammatory biomarkers, forming a nexus particularly pertinent to PCa. Notably, the transference of obesity-related traits to progeny accentuates concerns about the potential inheritance of these traits, which, in turn, may propel the onset of obesity-related comorbidities, notably PCa. This looming apprehension accentuates the imperative to fathom the intricate facets of associated factors and underlying mechanisms that drive this intricate interplay.

## Data Availability

No new data were created or analyzed in this study. Data sharing is not applicable to this article.
